# Exploration of the Tumor-Suppressive Immune Microenvironment by Integrated Analysis in *EGFR*-Mutant Lung Adenocarcinoma

**DOI:** 10.3389/fonc.2021.591922

**Published:** 2021-05-31

**Authors:** Teng Li, Xiaocong Pang, Junyun Wang, Shouzheng Wang, Yiying Guo, Ning He, Puyuan Xing, Junling Li

**Affiliations:** ^1^ Department of Medical Oncology, National Cancer Center/National Clinical Research Center for Cancer/Cancer Hospital, Chinese Academy of Medical Sciences and Peking Union Medical College, Beijing, China; ^2^ Department of Pharmacy, Peking University First Hospital, Beijing, China; ^3^ CAS Key Laboratory of Genome Sciences and Information, Beijing Institute of Genomics, Chinese Academy of Sciences/China National Center for Bioinformation, Beijing, China; ^4^ Research Institute, GloriousMed Clinical Laboratory (Shanghai) Co., Ltd., Shanghai, China

**Keywords:** immune microenvironment, lung adenocarcinoma, epidermal growth factor receptor (EGFR) mutation, Bioinformatics & Computational Biology, myeloid dendritic cells (mDCs), cytotoxic T lymphocyte (CTL)

## Abstract

**Background:**

Clinical evidence has shown that few non-small cell lung cancer (NSCLC) patients with epidermal growth factor receptor (*EGFR*) mutations can benefit from immunotherapy. The tumor immune microenvironment (TIME) is a significant factor affecting the efficacy of immunotherapy. However, the TIME transformational process in *EGFR*-mutation patients is unknown.

**Methods:**

The mRNA expression and mutation data and lung adenocarcinoma (LUAD) clinical data were obtained from The Cancer Genome Atlas (TCGA) database. Profiles describing the immune landscape of patients with *EGFR* mutations were characterized by differences in tumor mutation burden (TMB), ESTIMATE, CIBERSORT, and microenvironment cell populations-counter (MCP-counter).

**Results:**

In total, the TCGA data for 585 patients were analyzed. Among these patients, 98 had *EGFR* mutations. The TMB was lower in the *EGFR* group (3.94 mut/Mb) than in the *KRAS* mutation group (6.09 mut/Mb, *P* < 0.001) and the entire LUAD (6.58 mut/Mb, *P* < 0.001). The *EGFR* group had a lower population of activated immune cells and an even higher score of immunosuppressive cells. A further inter-group comparison showed that differences in the TMB and tumor-infiltrating lymphocytes were only found between patients with oncogenic mutations and unknown mutation. Meanwhile, there were more myeloid dendritic cells (DCs) in *EGFR* 19del than in L858R-mutation patients and in common mutation patents than in uncommon mutation patients (*P* < 0.05). Additionally, we established a D score, where D = MCP-counter score for cytotoxic T lymphocytes (CTLs)/MCP-counter score for myeloid DCs. Further analysis revealed that lower D scores indicated immune suppression and were negatively related to several immunotherapy biomarkers.

**Conclusions:**

The TIME of *EGFR* mutant NSCLC was immunosuppressive. Myeloid DCs gradually increased in *EGFR* 19del, L858R, and uncommon mutations. The potential role of CTLs and DCs in the TIME of patients requires further investigation.

## Introduction

Lung cancer is one of the leading causes of cancer mortality worldwide (1). Nearly 85% of patients with lung cancer are diagnosed with non-small-cell lung cancer (NSCLC). The two main histological subtypes of NSCLC are adenocarcinoma (ADC) and squamous cell carcinoma (SCC). However, ADC and SCC show different characteristic according to the mutation landscape at the molecular level. As reported, the epidermal growth factor receptor (*EGFR*) mutation is one of the most common mutated genes detected in adenocarcinoma. Previous studies have shown that *EGFR* mutations occurred more frequently in females, non-smokers, and Asian patients, and the majority of *EGFR* mutations were deletions in exon 19 or the L858R substitution in exon 21. Other mutations located in exons 18 and 20 are rare but can also cause *EGFR* gene activation (2).

For NSCLC patients harboring a sensitizing *EGFR*-mutation, treatment with *EGFR* tyrosine kinase inhibitors (TKIs) leads to longer progression-free survival (PFS) and has already become the first-line treatment. At present, three generations of *EGFR* TKIs are globally available for the treatment of NSCLC and have significantly improved the prognosis of patients (3–5). However, a significant portion of patients with co-mutations or rare mutations of *EGFR* gain little benefit from TKIs treatment (6). Additionally, almost all patients treated with TKIs eventually experience tumor relapse and acquire resistance. For such patients, the use of TKIs combined with chemotherapy or a monoclonal anti-vascular endothelial growth factor antibody has become the treatment of choice (7). However, the efficacy remains unsatisfactory, and there is still a great need for new treatment strategies.

In recent years, clinical trials have provided unequivocal evidence of the efficacy of immune checkpoint inhibitors (ICIs), so they have become a standard therapy in advanced NSCLC. Still, a major limitation was that patients with sensitizing *EGFR* mutations were excluded from most clinical trials. A meta-analysis demonstrated that ICIs prolonged overall survival in the EGFR wild-type subgroup [hazard ratio, (HR), 0.67], but not in the *EGFR* mutant subgroup (HR, 1.11), and prolonged overall survival in the *KRAS*-mutant subgroup (HR, 0.65), but not in the *KRAS* wild-type subgroup (HR, 0.86) (8). Currently available clinical trial data have shown that single-agent immunotherapy and in combination with TKIs are inappropriate for *EGFR* mutant patients (9). Thus, it is challenging to identify potential patients who could benefit from ICIs and to help patients with specific mutations benefit from immunotherapy.

**Figure 1:**
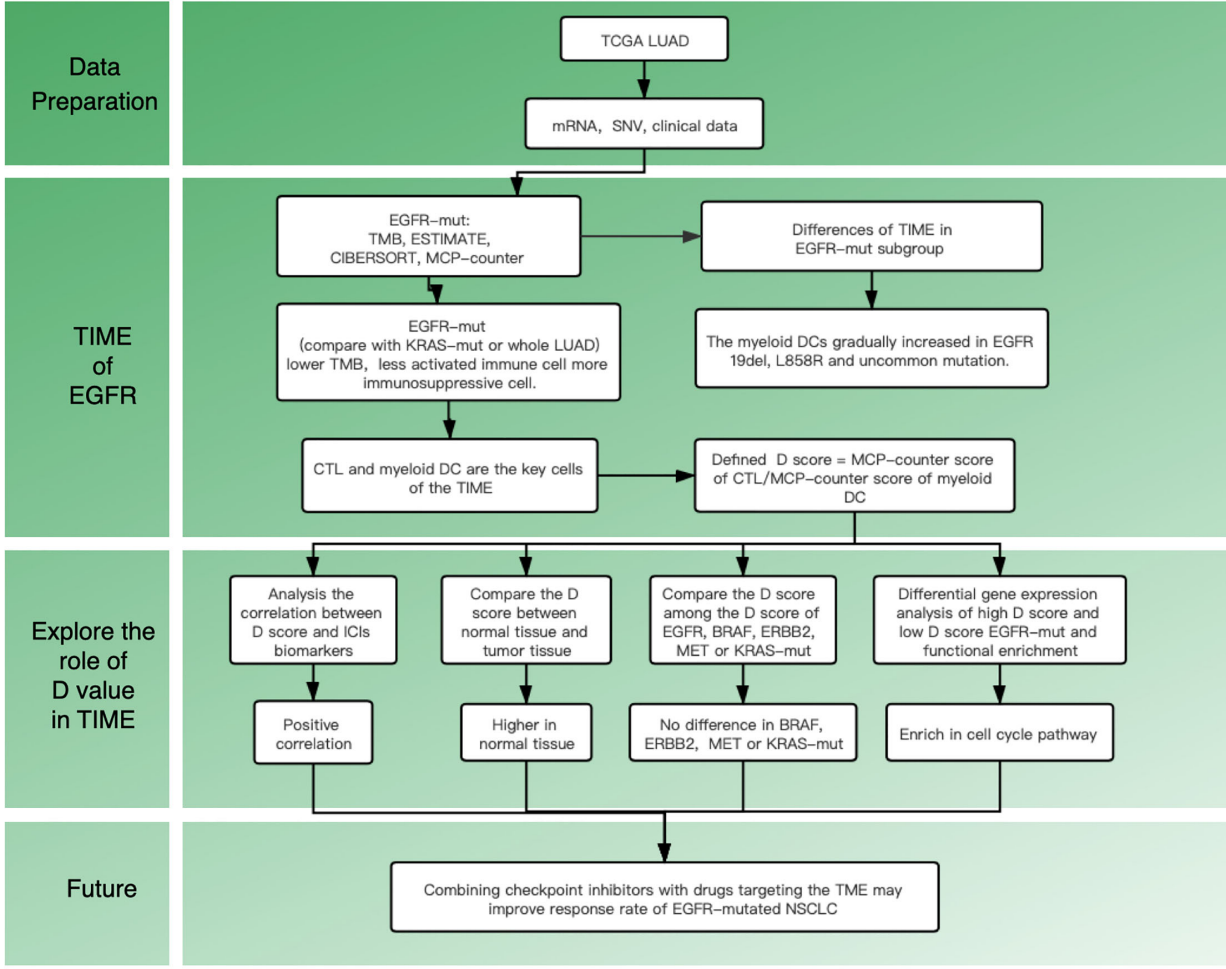
Flowchart of the study design.

Recent studies on the tumor immune microenvironment (TIME) suggest that the abundance and location of tumor-infiltrating lymphocytes (TILs) can be a potential predictor of response to ICIs (10). Clinical studies also have confirmed that reversing the TIME might reduce tumor-induced immunosuppression in patients with a mutated *EGFR* (11). All of these studies suggest that we should pay attention to the TIME of mutant lung cancer and seek a breakthrough in treatment. Till now, the immune landscape remains unclear in *EGFR* mutant patients, especially for different mutant subtypes. Therefore, this study aims to explore the TIME in *EGFR* ^-^ *^mt^* adenocarcinoma and investigate the specific TIME features within different subgroups. The flow chart of this study is shown in **Figure 1**.

## Methods

### Patient Cohort and Genomic Data Processing

The Cancer Genome Atlas (TCGA) lung adenocarcinoma (LUAD) mRNA expression data, mutation data, and clinical information were downloaded from TCGA (http://cancergenome.nih.gov/) and the University of California at Santa Cruz Xena (UCSC Xena; http://xena.ucsc.edu/). For transcriptome data, raw counts and Fragments Per Kilobase of transcripts per Million mapped reads [FPKM] were acquired. The FPKM was then transformed to TPM for further calculation. Genomic alteration was downloaded from the cBioPortal for Cancer Genomics (cBioPortal; http://cbioportal.org).

Common *EGFR* mutations were defined as exon 19 deletion and exon 21 L858R without exon 20 T790M. Uncommon *EGFR* mutations were defined as other oncogenic mutations, such as G719X, S768I, and L861Q in exons 18, 20, and 21, respectively. Except as noted above, others that cannot lead to *EGFR* activation were defined as unknown mutations. Patients with co-mutations of different *EGFR*-mutation status were excluded from all groups.

### Analysis of Immune Landscape

Tumor mutational burden (TMB): The TMB was defined as the total number of somatic mutations per megabase (Mb) of the genome examined. The normalized TMB = (whole exome non-synonymous mutations)/(38 Mb).

Estimation of STromal and Immune cells in MAlignant Tumor tissues using Expression data (ESTIMATE): ESTIMATE (12) was used to assess the immune infiltration (ImmuneScore), stromal content (StromalScore), and combined (ESTIMATEScore) of the samples. This kind of scoring can be used to estimate tumor purity. ESTIMATE was downloaded from the MD Anderson Cancer Center database (https://bioinformatics.mdanderson.org).

CIBERSORT: CIBERSORT (13) is an analytical tool that applies a deconvolution algorithm to estimate the proportions of 22 leucocyte subtypes based on RNA-seq data. Results with a CIBERSORT *P*-value of <0.05 were used for subsequent calculation. The package ‘CIBERSORT’ was used to calculate the percentage.

Microenvironment cell populations-counter (MCP-counter): MCP-counter (14) is a computational Method based on the mean marker gene expression that is specifically expressed in the cell type. The eight immune-cell lineage scores were estimated by using the R package MCP-counter algorithm.

### Differentially Expressed Genes and Functional Pathways Analysis

Differential gene expression was determined by using the EdgeR software package. Gene Ontology (GO) and Kyoto Encyclopedia of Genes and Genomes (KEGG) enrichment (false discovery rate < 0.05, Foldchange > 1) analysis of differentially expressed genes (DEGs) were performed to search for biological functions and pathways.

### Statistical Analysis

The statistical analysis of tumor purity and of the presence of infiltrating stromal/immune cells in tumor tissues was performed by using R packages: MCP-counter and CIBERSORT. Statistical comparisons were evaluated by using the Wilcoxon rank-sum test and Kruskal–Wallis tests. The clinicopathological characteristics were compared by using chi-square test. Correlations were assessed by using Pearson correlational distances. A two-sided *P*-value of <0.05 was considered to be indicative of statistical significance.

## Results

### Clinical Characteristics of the Patients

In total, 585 patients data were downloaded from the TCGA data set. Among these, 98 cases involved *EGFR*-mutation. Then, the patients were divided into four groups according to their *EGFR*-mutation type (19del, L858R, uncommon mutation, unknown mutation group). Clinical data were available for 68 of the 98 patients. Baseline characteristics are presented in **Table 1**.

**Table 1:** Demographic characteristics of the patients at baseline.

Characteristics	Type	Total	19del	L858R	Uncommon mutation	Unknown mutation
Age	<=65	33 (48.53%)	11 (42.31%)	9 (50%)	7 (58.33%)	6 (50%)
	>65	32 (47.06%)	12 (46.15%)	9 (50%)	5 (41.67%)	6 (50%)
	Unknown	3 (4.41%)	3 (11.54%)	0 (0%)	0 (0%)	0 (0%)
Gender	FEMALE	49 (72.06%)	22 (84.62%)	15 (83.33%)	9 (75%)	3 (25%)
	MALE	19 (27.94%)	4 (15.38%)	3 (16.67%)	3 (25%)	9 (75%)
Stage	Stage I–II	52 (76.47%)	19 (73.08%)	15 (83.33%)	9 (75%)	9 (75%)
	Stage III–IV	15 (22.06%)	7 (26.92%)	3 (16.67%)	3 (25%)	2 (16.67%)
	Unknown	1 (1.47%)	0 (0%)	0 (0%)	0 (0%)	1 (8.33%)
T	T1–2	58 (85.29%)	26 (100%)	15 (83.33%)	9 (75%)	8 (66.67%)
	T3–4	9 (13.24%)	0 (0%)	3 (16.67%)	2 (16.67%)	4 (33.33%)
	TX	1 (1.47%)	0 (0%)	0 (0%)	1 (8.33%)	0 (0%)
M	M0	47 (69.12%)	17 (65.38%)	15 (83.33%)	7 (58.33%)	8 (66.67%)
	M1	3 (4.41%)	1 (3.85%)	0 (0%)	1 (8.33%)	1 (8.33%)
	Unknown	18 (26.47%)	8 (30.77%)	3 (16.67%)	4 (33.33%)	3 (25%)
N	N0	42 (61.76%)	12 (46.15%)	12 (66.67%)	7 (58.33%)	11 (91.67%)
	N1-3	23 (33.82%)	13 (50%)	6 (33.33%)	3 (25%)	1 (8.33%)
	Unknown	3 (4.41%)	1 (3.85%)	0 (0%)	2 (16.67%)	0 (0%)

### TIME of the Patients With *EGFR* Mutations

**Figure 2:**
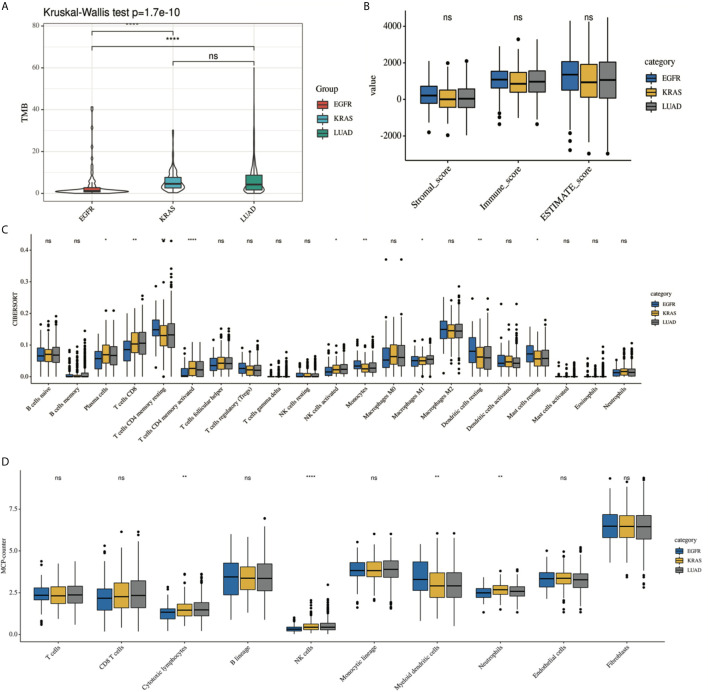
Comparison of the immune landscape of EGFR-mutated group, KRAS-mutated group and the whole lung adenocarcinoma. The TIME of EGFR-mutated NSCLC show an immunosuppressive status with lower **(A)** TMB, a lower score of activated immune cell and an even higher score of immunosuppressive cell, estimated by **(C)** CIBERSORT and **(D)** MCP-counter. No difference was found in tumor purity among three group **(B)**. *P < 0.05, **P < 0.01, and ***P < 0.0001, respectively. ns, not significant.

**Supplementary Figure 1** shows the immune landscape of the *EGFR* mutant group. For further evaluation, we compared the TIME in the *EGFR* group with that in the *KRAS* group, and the whole LUAD. The results showed that the TMB was lower in the *EGFR* group (3.94 mut/Mb) than in the *KRAS* group (6.09 mut/Mb, *P* < 0.001) and in the whole LUAD (6.58 mut/Mb, *P* < 0.001) (**Figure 2A**). For tumor purity, which was inferred from the ESTIMATE purity score, there were no significant differences between the three groups (*P* > 0.05) (**Figure 2B**). For TILs, the *EGFR* group had a lower score of activated immune cells (CD8 T cells, cytotoxic lymphocytes (CTLs), NK cells) and an even higher score of immunosuppressive cells [myeloid dendritic cells (DCs)] according to the MCP-counter results than *KRAS* group and the whole LUAD (**Figure 2D**, *P* < 0.05). The same trend was observed through analysis of the immune-cell proportion by using the CIBERSORT deconvolution Method (**Figure 2C**. *P* < 0.05).

### Further Analysis of the TIME in the *EGFR* Mutations Subgroup

**Figure 3:**
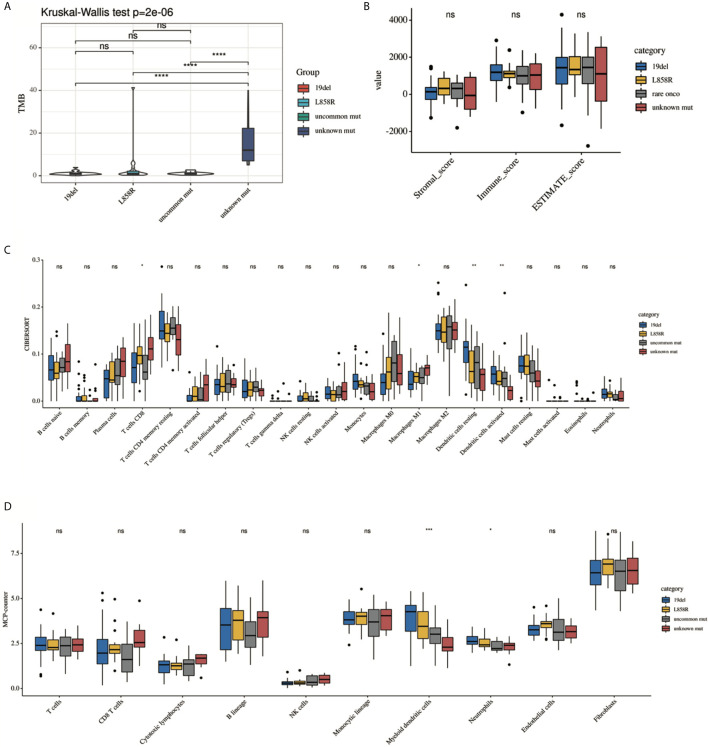
Comparison of the immune landscape among 19del, L858R, uncommon mutation and unknown mutation group. **(A)** TMB, **(B)** ESTIMATE, **(C)** CIBERSORT, **(D)** MCP-counter. Only significant difference in myeloid DC cells was presented among different groups estimated by **(C)** CIBERSORT and **(D)** MCP-counter. *P < 0.05, **P < 0.01, ***P < 0.001 and ****P < 0.0001, respectively. ns, not significant.

Previous studies have suggested that the efficacy of immunotherapy was different among different mutant subtypes. We hypothesized that this may be related to TIME. We then conducted a subgroup analysis to further explore the differences in TIME among different *EGFR* subgroups. Analysis showed that there were significant differences among the four subgroups (19del, L858R, uncommon mutations, unknown mutations) in the TMB, CD8 T cells, and DCs (**Figure 3**). However, there were no differences in the purity scores among the four groups (by ESTIMATE).

To determine the source of the differences, we first classified the *EGFR*-mutation patients into oncogenic and unknown mutation groups according to whether EGFR was activated. The oncogenic group showed a lower TMB, a lower fractions of activated immune subpopulations (by CIBERSORT), and a high abundance of myeloid DCs (by MCP-counter) (**Supplementary Figure 2**, *P* < 0.05). Second, oncogenic *EGFR* mutations can be divided into common and uncommon mutations. There were more myeloid DCs in the common mutations group than in the uncommon mutations group (by MCP-counter). No statistical differences in TMB, tumor purity or fractions of the most immune subgroup were observed between this two groups (**Supplementary Figure 3**, *P* < 0.05). Thirdly, as mentioned above, common *EGFR* mutations include exon 19 deletion and exon 21 L858R. The analysis results showed that there were also no differences in the TMB and ESTIMATE scores between this two subgroups. But a trend toward an immunosuppressive environment was observed in the 19del group. The proportion of resting DCs in the 19del group showed a significant decrease than that in the L858R group (by CIBERSORT, *P* < 0.05) (**Supplementary Figure 4**).

### Define a Score (D) Based on the MCP-Counter

Subgroup analysis of clinical trials showed that the efficacy of tumor immunotherapy was better in the KRAS group than in the EGFR group. To further characterize which types of infiltrating cells have important roles in immunotherapy, the compositions of TILs were compared between the *KRAS* and *EGFR* groups. Analysis by MCP-counter (as well as the CIBERSORT results) showed that more abundant CTLs and a lower population of myeloid DCs were observed in the *KRAS^-mt^* group than in the *EGFR^-mt^* group (**Supplementary Figure 5**). The results above suggest that CTLs and myeloid DC have an essential role in TIME. According to this result, we built a simplified model (D score) to quantify the generalized TIME state and defined a D score as D = MCP-counter score of CTLs/MCP-counter score of myeloid DCs. The lower the D score, the greater degree of immunosuppression in the TIME. To test the performance of D score, we performed the following analysis.

### Functional Enrichment Analysis in Different *EGFR* Mutant Groups Defined by the D Score

**Figure 4:**
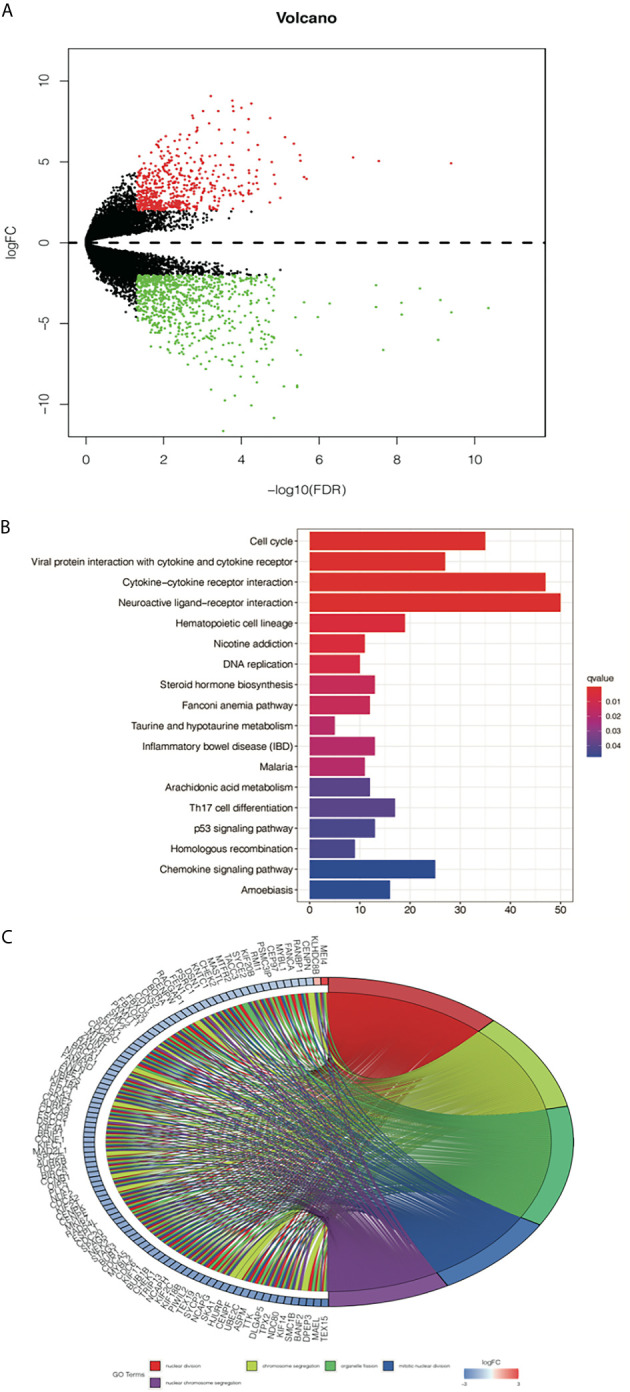
Analysis of differentially expressed genes (DEGs) from the D value of EGFR-mut group quartile 1 compared with quartile 3. **(A)** Volcano plot showing DEGs between higher D score group and lower D score group. **(B)** Top 10 enrichments of up-regulated DEGs by KEGG analyses. **(C)** Gene Ontology analyses of DEGs according to their biological process.

To further study the differences in gene expression between different D values, *EGFR*-mutation patients were grouped by D value quartiles, with quartile 1 having the lowest and quartile 3 the highest D level (n = 16 in each group). Differentially expressed genes between the two groups were identified by performing an EdgeR analysis. Among the DEGs identified, 890 were upregulated, and 1373 were downregulated. The DEGs are shown as a volcano plot in **Figure 4A**. The candidate DEG functions and signaling pathway enrichment were analyzed by using GO terms and the KEGG pathway. Overall, 18 KEGG pathways were significantly enriched. The top five KEGG pathways were the cell cycle, viral protein interaction with cytokine and cytokine receptors, cytokine–cytokine receptor interaction, neuroactive ligand–receptor interaction, and hematopoietic cell lineage (**Figure 4B**). The organelle fission GO term was the top GO term, followed by nuclear division, receptor ligand activity chromosome segregation, and chromosomal region (**Figure 4C**).

### Exploration of D Score Among Patients With Different Driver Mutation

**Figure 5:**
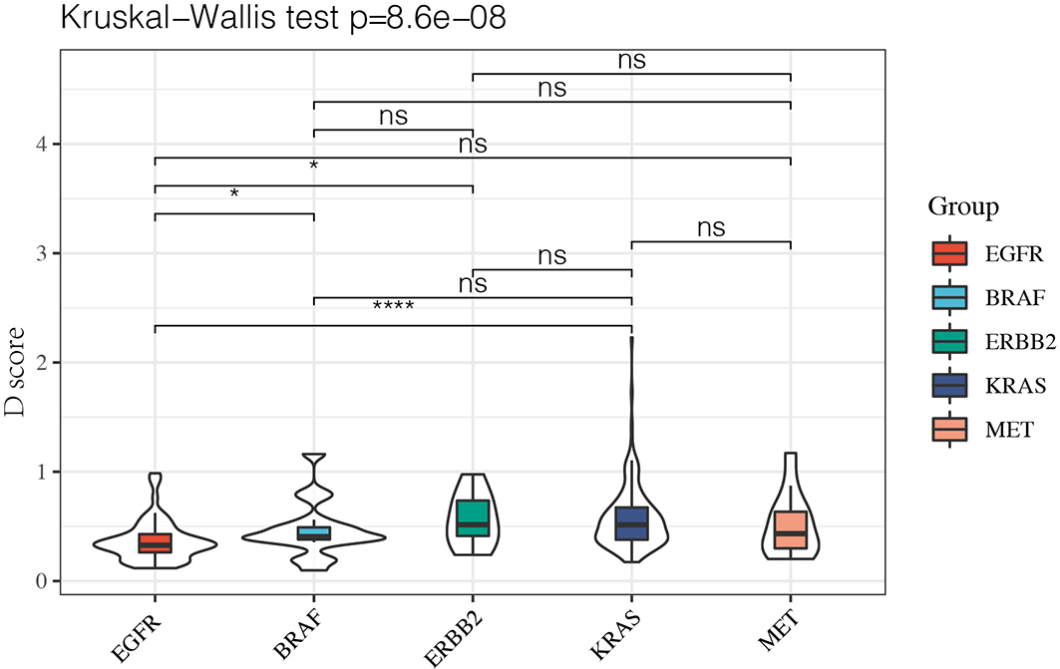
The D value of EGFR group was lower than rare mutation group except MET mutation and there was no significant difference among BRAF, ERBB2, KRAS and MET mutation. *P < 0.05 and ****P < 0.0001, respectively. ns, not significant.

Since it remained uncertain whether NSCLC with targetable drivers will benefit from immunotherapy, we further calculated the D value of different mutation subgroups, including *EGFR*, *BRAF*, *ERBB2*, *KRAS*, and *MET* mutations. The results showed that the average D values were 0.37, 0.47, 0.56, 0.57, and 0.51 in patients with EGFR, *BRAF*, *ERB*B2, *KRAS*, and *MET* mutations. Inter-group analysis showed that the D value was lower in the *EGFR* group than in the other rare mutation groups (*P* < 0.05) except for the *MET* mutation. However, there were no significant differences in the D values among the *BRAF*, *ERBB2*, *KRAS*, and *MET* mutations (**Figure 5**).

### Clinical Characteristics and Immune-Cell Infiltration of Different D score Groups in LUAD

**Figure 6:**
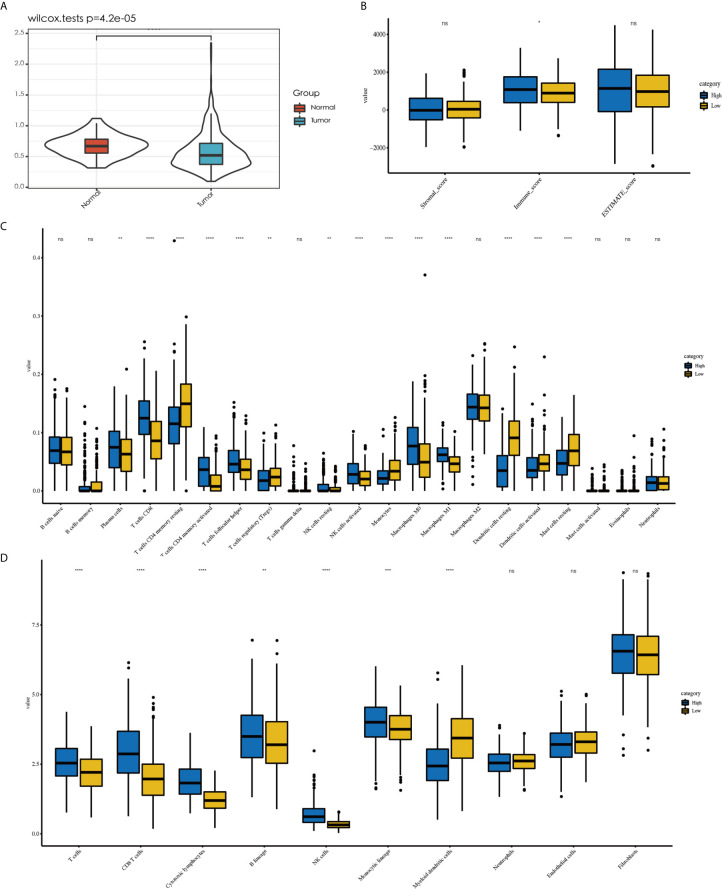
Explore the role of D value in LUAD. Tumor tissue showed a lower D value than normal tissue **(A)**. No difference in tumor purity was observed between high-D and low-D group **(B)**. Less abundant of activated immune cell were observed in low-D group by CIBERSORT **(C)** as well as the result MCP-counter **(D)**. *P < 0.05, **P < 0.01, ***P < 0.001 and ****P < 0.0001, respectively. ns, not significant.

To examine the significance of the D score, we further studied its role among all TCGA–LUAD patients. We first compared the D score between tumors and normal tissues from TCGA. The results showed that the D value was higher in normal tissue than in tumor tissue, which was consistent with expectations (**Figure 6A**, *P* < 0.001). We then compared the clinicopathological features and immune landscape characteristics of different LUAD patients by their D scores. For further analysis, the median D score was selected as the cutoff value. The patients were divided into two groups: the high (D > 0.51, n = 252) group and low (D ≤ 0.51, n = 251) D group. We found there were no significant differences in the D scores of the samples among the clinicopathological features groups, including T, N, M, Stage, Grade, and Age (*P* > 0.05) (**Supplementary Table 1**). We also observed that the tumor purity difference between the patients in the high- and low-D groups was not significant (median ESTIMATE score, *P* > 0.05). However, the immunescore was higher for the high-D group, as expected (*P* < 0.05) (**Figure 6B**).

Because the D score reflected the immunosuppressive environment based on T cells and DCs, we decided to explore whether the levels of other immune cells in different D groups were consistent with expectations. The different immune cell distribution between the high- and low-D groups is shown in **Figures 6C, D**. In addition to CD8+T cells and DCs, we observed that the high-D group had higher infiltration of activated T cells CD4 memory activated, T- cells follicular helper, NK cells activated, and macrophages M1 according to CIBERSORT (*P* < 0.05). In contrast, the low-D group had higher infiltration of Ts cells CD4 memory resting, T cells regulatory (Tregs), and monocytes and mast cells resting (*P* < 0.05) (**Figure 6C**). Similar to this result, we found that a variety of T-cell MCP-counter scores decreased in the low-D group (*P* < 0.05) (**Figure 6D**).

### Relationship Between the D Score and Predictive Biomarkers for Immunotherapy

**Figure 7:**
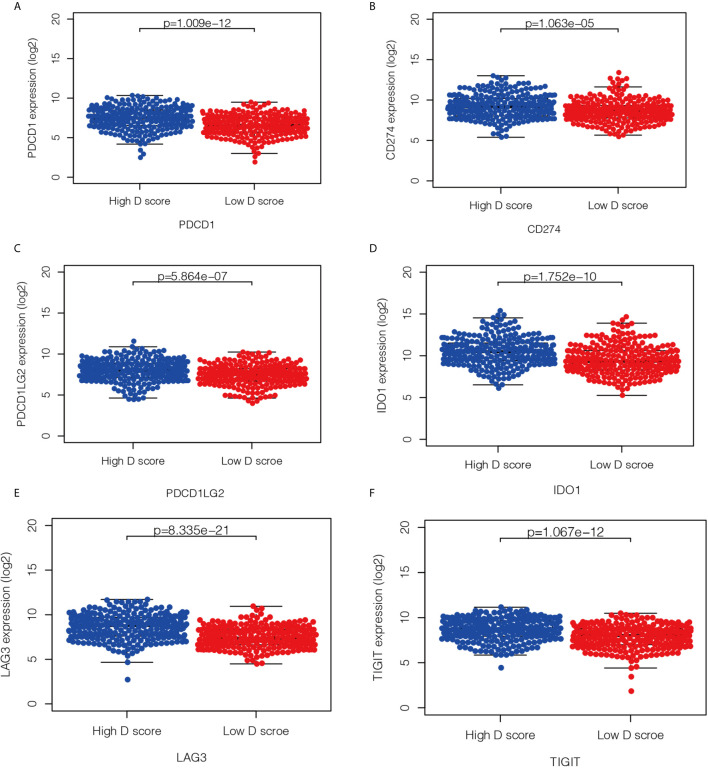
Relationship between the D score and the expression of immune checkpoint. **(A–F)** the distribution of mRNA level of PDCD1, CD274, PDCD1LG2, LAG3, TIGIT and IDO1 in high- and low-D score groups.

Immunotherapy is currently a first-line treatment for lung adenocarcinoma. However, it remains challenging to identify immune resistance and the beneficiary groups. To explore the predictive role of the D value in immunotherapy, we further explored the relationship between the D score and predictive biomarkers for ICIs. First, we found a positive correlation between D scores and TMB (r = 0.30, *P* < 0.001), which suggests that the lower the D value, the lower the TMB, and the less likely it is to benefit from immunotherapy. In addition, we further explored the association between different patient groups defined by the D score and immune checkpoints. We found that lower mRNA expression levels of PDCD1, CD274, PDCD1LG2, LAG3, TIGIT, and IDO1 were observed in the low-D score patients (**Figures 7A–F**, *P* < 0.05). The results also suggest that a lower D value is a good indicator of immunosuppression and a potentially negative biomarker for immunotherapy.

## Discussion

**Figure 8:**
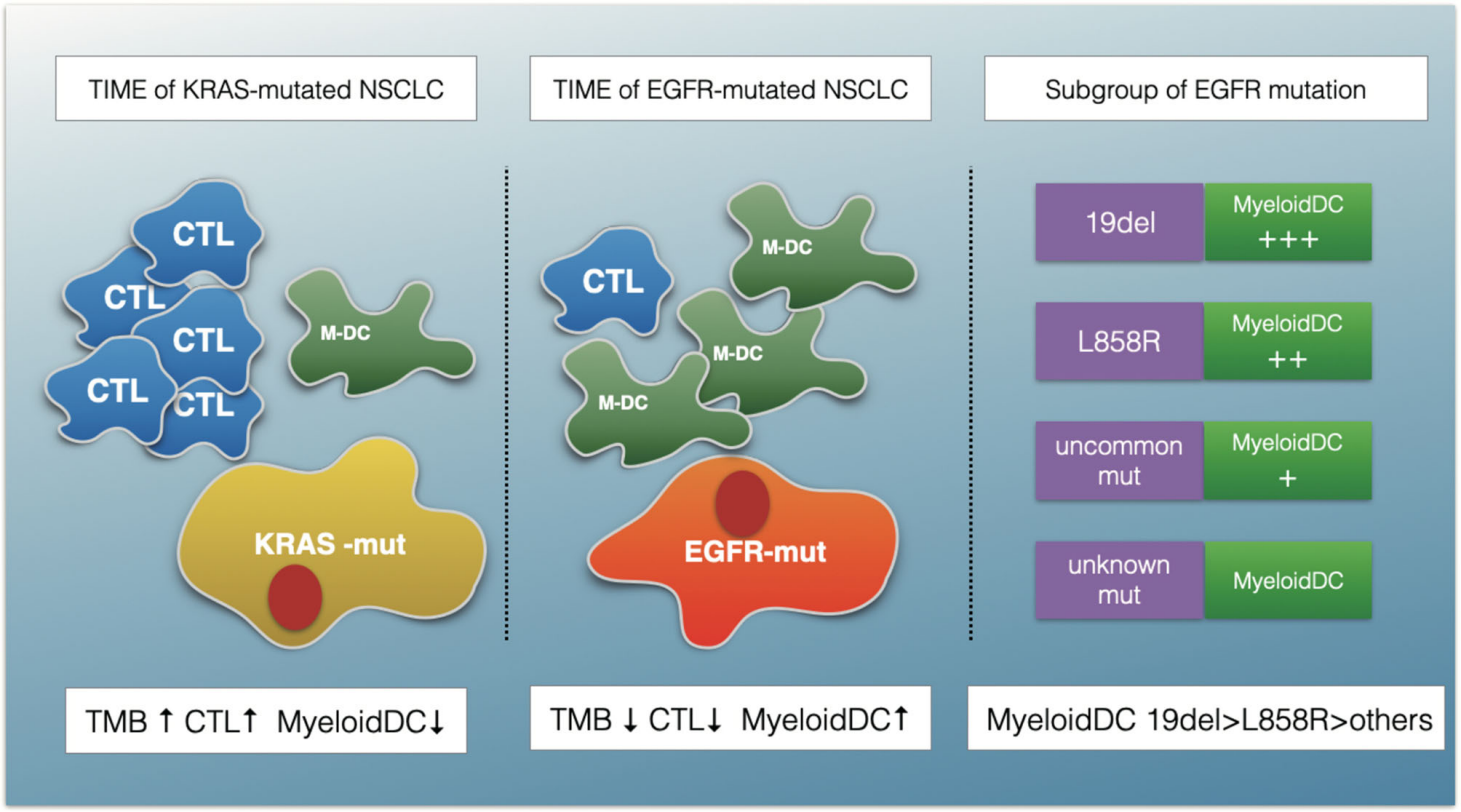
A schematic summarizing the major findings of this study. The TIME of EGFR-mutated NSCLC show an immunosuppressive status with lower TMB, fewer cytotoxic T cells (CTLs) and more myeloid DC (M-DC) cells compared with KRAS-mutated NSCLC. The myeloid DCs increased in EGFR 19del compared with L858R mutation patients, and in common mutation patents compared with uncommon mutations.

Although ICIs have demonstrated clinical activity in a variety of tumors, resistance of cancer to ICI therapy remains a major clinical problem (15). From a clinical perspective, resistance to PD-(L)1 inhibitors can be classified into three distinct scenarios: primary resistance, secondary resistance, and progression after treatment discontinuation (16). The mechanisms of primary resistance to immunotherapy include tumor-cell intrinsic (for example, absence of antigenic proteins) and tumor-cell extrinsic (for example, absence of T cells and infiltration of immunosuppressive cells) (17), which can interact with each other. Increasing evidence has suggested that identifying immunophenotyping will help predict outcomes of immunotherapy and provide a novel direction for overcoming resistance (18, 19). On the basis of the above, we conducted the present study. **Figure 8** is a schematic representation of the findings presented in this article.

In this study, we characterized four aspects to describe the immune landscape of patients with *EGFR* mutations. The first aspect is the TMB. Previous studies have shown that the TMB is an important criterion for successful immunotherapy. Our findings suggest that the TMB was lower for the *EGFR* group than for the *KRAS* group and the whole LUAD. In an open-label, randomized, phase 3 trial (CheckMate 227), the researchers demonstrated longer PFS time in NSCLC patients with a high TMB (defined as ≥10 mut/Mb), regardless of PD-L1 expression or tumor type (HR, 0.58; 95% CI, 0.41–0.81) (20). However, in our study, only 12.5% of the *EGFR* patients had TMB of >10mut/Mb. Seventy-eight percent of patients in the *EGFR* group had TMB of <5mut/Mb. This finding partly explains the reason for the poor curative effect in the *EGFR^-mt^* group. The second aspect is the ESTIMATE score. The higher the ESTIMATE score, the lower the tumor purity. Our study showed that there were no significant differences in tumor purity among the three groups, which was inferred by using the ESTIMATE purity score. The third and fourth aspects were CIBERSORT and MCP-counter. Both of these tools are mathematical Methods to estimate TILs. A previous study suggested that immune-cell infiltration was associated with antitumor activity. It has been reported that high stromal infiltration of CD8+ ICs and CD4+ ICs was associated with better overall survival by analyzing 139 nivolumab-treated NSCLC simple tumor tissue specimens (21). In our study, compared with KRAS and the whole LUAD group, the TIME in the *EGFR* mutant patients showed a trend of diminishing activated TILs and increasing numbers of immunosuppressive cells. Some previous experimental studies have reported similar results. Although there are artificial intelligence approaches to calculate the absolute count of immune cells, these Methods and tools are still evolving. At present, the most commonly used Method to quantify the number and type of immune cells is by immunohistochemistry (IHC). A study based on 245 Chinese NSCLC patients showed that lower immune infiltration was associated with *EGFR* mutations in adenocarcinoma samples through IHC staining of CD8 (22). Similarly, a French study also confirmed this conclusion; the researchers found that the expression of PD-L1 was decreased, and the density of CD8+T lymphocytes was lower in patients with *EGFR* mutations in lung cancer through IHC. In addition, with the development of technology in recent years, some new technologies, such as Digital Spatial Profiling, have also gradually shown the advantages of quantifying the tumor microenvironment, but it has not been widely used (23). Clinically, our results are also consistent with clinical evidence showing that compared with *KRAS*-mutant patients, patients with *EGFR* mutations-mutant are difficult to rarely benefit from immunotherapy (8).

In the era of molecular targeted therapy, different subtypes of *EGFR* mutations may cause different TKIs sensitivity. However, it remains undetermined if there are differences in the TIMEs and ICI efficacies among different *EGFR*-mutation subtypes. In a registration study, a total of 125 *EGFR*-mutation patients treated with ICIs were included. That study showed that different *EGFR-*patient subgroups had different PFS and OS durations. The median PFS was 1.4 months for patients with T790 single or multiple mutations, 1.8 months for patients with the exon 19del mutation, 2.5 months for patients with the exon21 L858R mutation, and 2.8 months for patients with other mutations (*P* < 0.001) (24). In the present study, we also observed differences in the immune landscapes among the 19del, 21L85R, uncommon-mutant, and unknown-mutant groups. Through a further inter-group comparison, we found that differences in the TMB and TILs were only found between patients with oncogenic mutations and unknown mutations. No significant differences were observed in the activated immune cells between 19del patients and L858R-mutation patients or between common mutation and uncommon mutation patients. However, the myeloid DCs increased in the 19del group relative to those in the L858R group and in the common mutation group relative to those in the uncommon group. These findings indicated that, from an immunological perspective, oncogenic mutation might be an important factor for cellular immune suppression. Targeting DC therapy may be an interesting future direction for *EGFR*-mutation patients.

As is known, the tumor microenvironment is a complex and dynamic system formed by stromal, immune, and inflammatory cells and the extracellular matrix (ECM) (18). In our research, to further identify the key immune cells in the TIME, we analyzed the TILs in the KRAS and *EGFR* groups, including CIBERSORT and MCP-counter results, and we finally determined through inter-group comparisons that activated T cells and resting DCs were the key analysis factors. Various studies have proven the significant functions of T cells in the antitumor process. The role of DCs has been gradually recognized in recent years. Previous studies have shown that DCs are central to the initiation of antigen-specific immunity and tolerance. In the TIME, DCs acquire, process, and present tumor-associated antigens on major histocompatibility complex molecules and provide co-stimulation and soluble factors to shape T-cell responses (25). For the above reasons, we defined D = MCP-counter score of CTLs/MCP-counter score of myeloid DCs. Considering that the MCP-counter score presents as the geoMETric mean of marker gene expression (26), the D value, as a further calculation of the MCP-counter score, might have good clinical application and promotional value by validation through multiple IHC analyses with tyramide signal amplification or DSP technology in the future.

An interesting result of D value analysis was that the differential genes were mainly enriched in the cell cycle pathway when the low-D-score group was compared with the high-D-score group for *EGFR*-mutation patients. Although previous clinical trials have shown that necitumumab, an anti-EGFR antibody combined with abemaciclib (a CDK4/6 inhibitor), did not produce an additive effect over single-agent activity in patients with stage IV NSCLC, no clinical studies have been conducted to explore the effectiveness of immunotherapy combined with a CDK4/6 inhibitor (22). Some basic studies have established that CDK4/6 inhibitors could enhance T-cell activation and induce a T-cell inflamed TME (23, 24). Based on our analysis results, the combination of ICIs with a cell cycle-related drug may be a potential therapeutic option for *EGFR*-mutation patients. However, this hypothesis needs to be confirmed.

Mutation features comprise an important signature of LUAD. In addition to classical mutations, some other mutations have gained increasing attention from researchers. Although ICIs have shown promising benefit in NSCLC, the efficacy of ICIs in NSCLC patients with rare mutations remains largely unknown. Previous reports have shown how different mutations affect the microenvironmental phenotype of tumors, which in turn affects the sensitivity of tumors to immunotherapy. For example, the TIME has been shown to have less immune-cell infiltration in *STK11*-mutation patients who have a worse prognosis after immunotherapy (27). A similar phenomenon was also observed in patients with *KEAP1* mutations, despite a high TMB level (28). Our data shows that the D scores of *BRAF*, *ERBB2*, *MET*, or *KRAS* were very similar, suggesting that they may have similar TIMEs in some ways. On the one hand, this result is consistent with previous real-world study data showing that ICI efficacies against *BRAF*-, *HER2*-, *MET*-, or *RET*-NSCLC patients were close to the efficacy observed in unselected NSCLC patients (29). On the other hand, our results support the application of ICIs from a TIME viewpoint.

Although immunotherapy has greatly improved the prognosis of patients with lung cancer, screening patients who are potentially effective or resistant to treatment remains challenging (30). Through early detection of potential biomarkers, we can choose individualized strategies for patients who may be resistant to immunotherapy, which may help to prolong the survival time (31). In our study, a lower D score was shown to be a new indicator of immunosuppression. This result was further verified by analyzing the TMEs of the high- and low-D groups in the whole LUAD population. Just as we expected, we observed less activated and more resting immune cells in the low-D group through analysis of immune cells. To further clarify the role of the D value in clinical practice, we further investigated the relationship between the D score and the well-studied immunotherapy predictive biomarkers. We found that low-D score patients had significantly lower levels of immune checkpoint gene expression and TMB. These novel findings suggest that the D score may be a predictive biomarker for immunotherapy response. This possibility needs to be confirmed in future prospective clinical studies.

In conclusion, the TIME of *EGFR* ^-^ *^mt^* NSCLC was found to be immunosuppressive. Myeloid DCs were higher in *EGFR* 19del patients than in L858R mutation patients and in common mutation patients than in uncommon mutations. CTLs and DCs have key roles in the TIME and may be potential predictors of immunotherapy efficacy. Certain aspects of the findings require further validation and qualification in a large longitudinal population study in the future.

## Data Availability Statement

Publicly available data sets were analyzed in this study. These data can be found here: portal.gdc.cancer.gov/.

## Author Contributions

Conception and design: TL and XP. Development of methodology: JW and YG. Analysis and interpretation of data (e.g., statistical analysis, biostatistics, computational analysis): NH and SW. Writing, review, and/or revision of the manuscript: TL and JW. Administrative, technical, or material support (i.e., reporting or organizing data, constructing databases): XP. Study supervision: PX and JL. All authors contributed to the article and approved the submitted version.

## Conflict of Interest

NH and JW were employed by the company GloriousMed Clinical Laboratory (Shanghai) Co., Ltd.

The remaining authors declare that the research was conducted in the absence of any commercial or financial relationships that could be construed as a potential conflict of interest.
