# Supplementary material for: Exploration of the Tumor-Suppressive Immune Microenvironment by Integrated Analysis in EGFR-Mutant Lung Adenocarcinoma
Source: Front Oncol. 2021 May 31;11:591922. doi: 10.3389/fonc.2021.591922 (PMC8200668; doi:10.3389/fonc.2021.591922)

Supplementary Figure 1 Four aspects to describe the immune landscape of patients with EGFR mutations : (A) TMB, (B) ESTIMATE, (C) CIBERSORT and (D) MCP-counter.

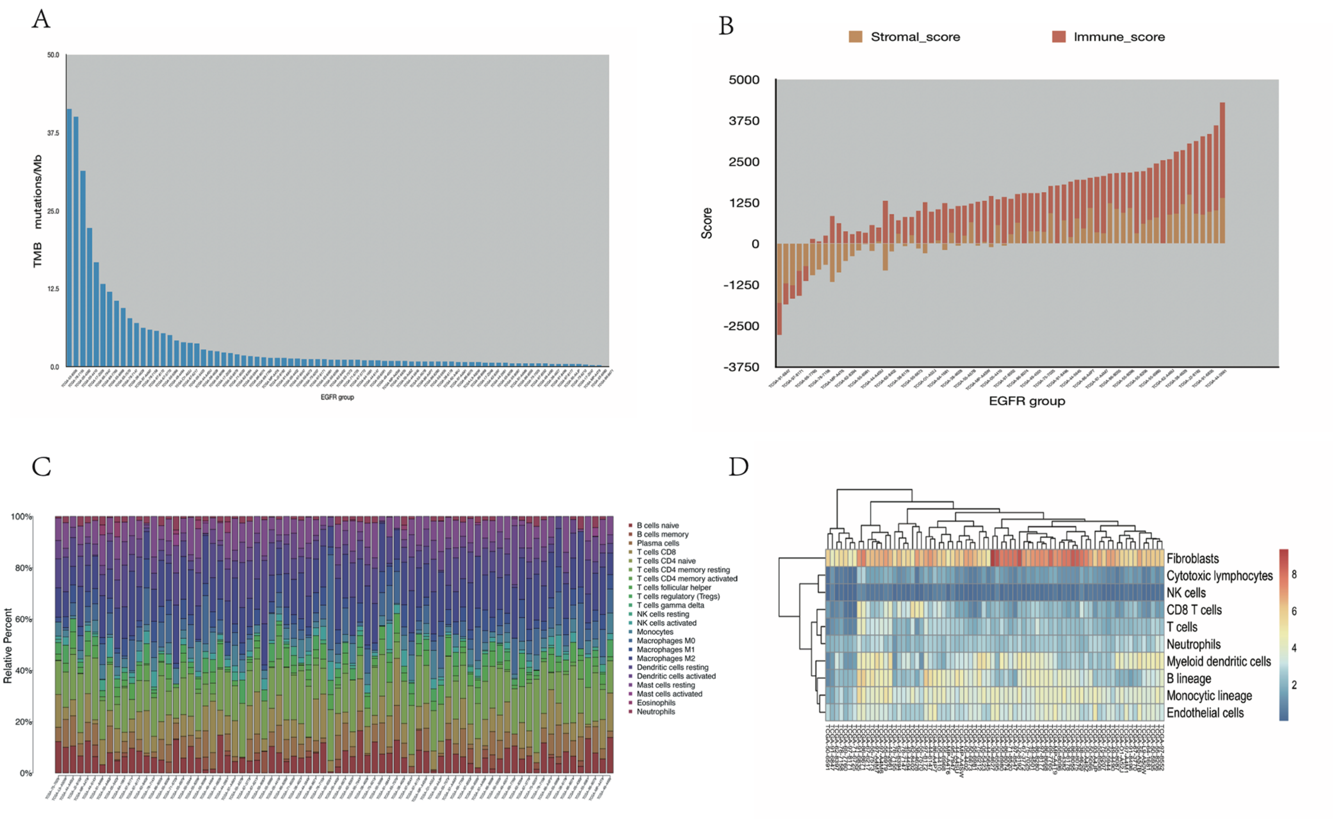

Supplement figure 2 Comparison of the immune landscape among EGFR 19del mutation and L858R mutation group. (A) TMB, (B) ESTIMATE, (C) CIBERSORT, (D) MCP-counter. The proportion of DC resting cells in Group 19del showed significant decrease compared with Group L858R (by (C) CIBERSORT) . *, **, ***, and **** represent P < 0.05, P < 0.01, P < 0.001 and P < 0.0001, respectively.

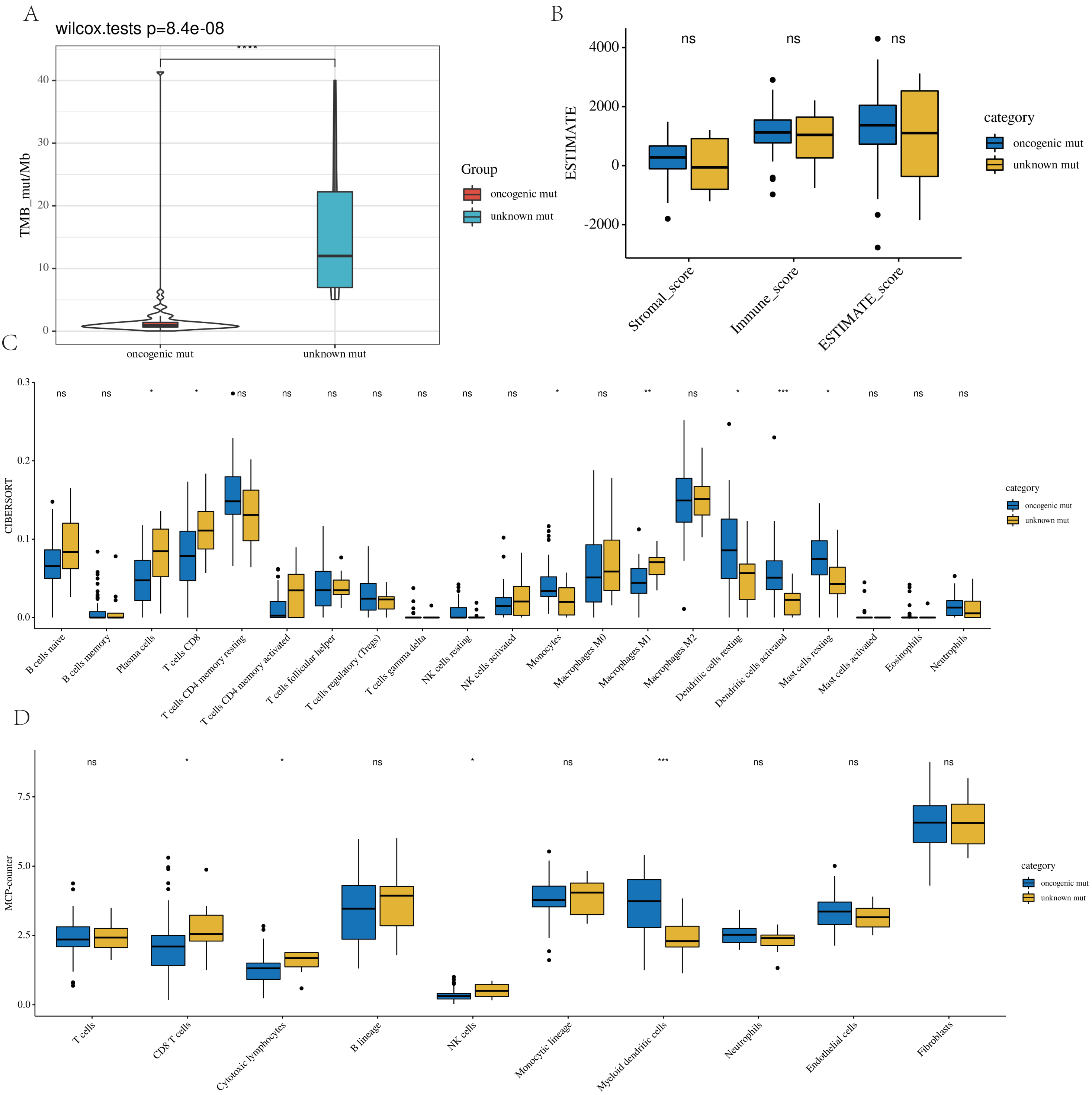

Supplement figure 3 Comparison of the immune landscape among EGFR common mutation group and uncommon mutation group.(A) TMB, (B) ESTIMATE, (C) CIBERSORT and (D) MCP-counter. No statistical difference was observed between the two groups in (A) TMB, (B) tumor purity or fractions of most immune subgroup between these two groups. But there were more myeloid DCs in common mutation group than in the uncommon mutation group (by (D) MCP-counter). *, **, ***, and **** represent P < 0.05, P < 0.01, P < 0.001 and P < 0.0001, respectively.

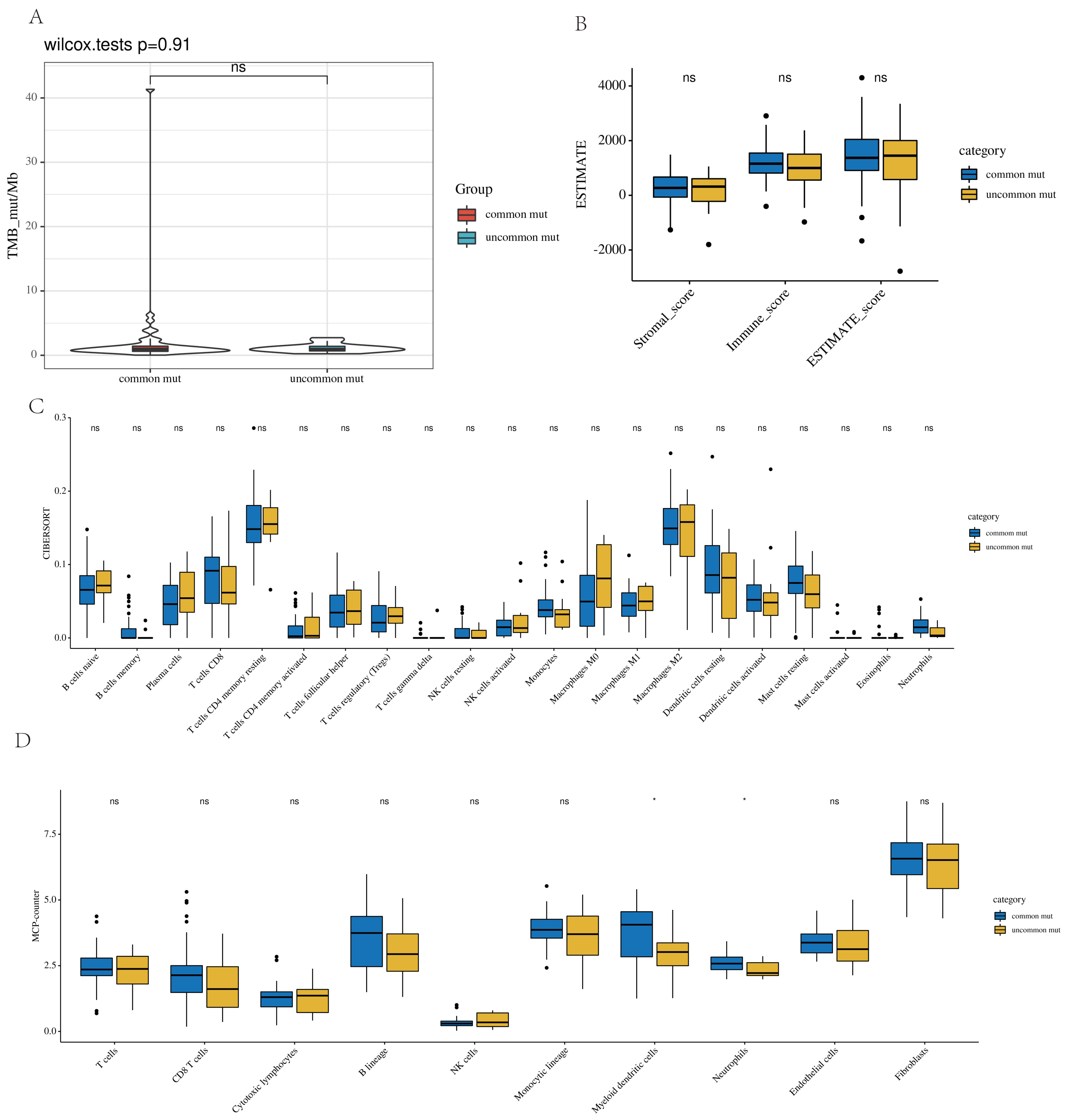

Supplement Figure 4 Comparison of the immune landscape among EGFR oncogenic mutation group and unknown mutation group.(A) TMB, (B) ESTIMATE, (C) CIBERSORT and (D) MCP-counter. Oncogenic group showed lower (A)TMB, lower fractions of activated immune subpopulations (by (C) CIBERSORT and high abundance of myeloid DC cells (by (D) MCP-counter). No difference was found in tumor purity between two groups (B). *, **, ***, and **** represent P < 0.05, P < 0.01, P < 0.001 and P < 0.0001, respectively.

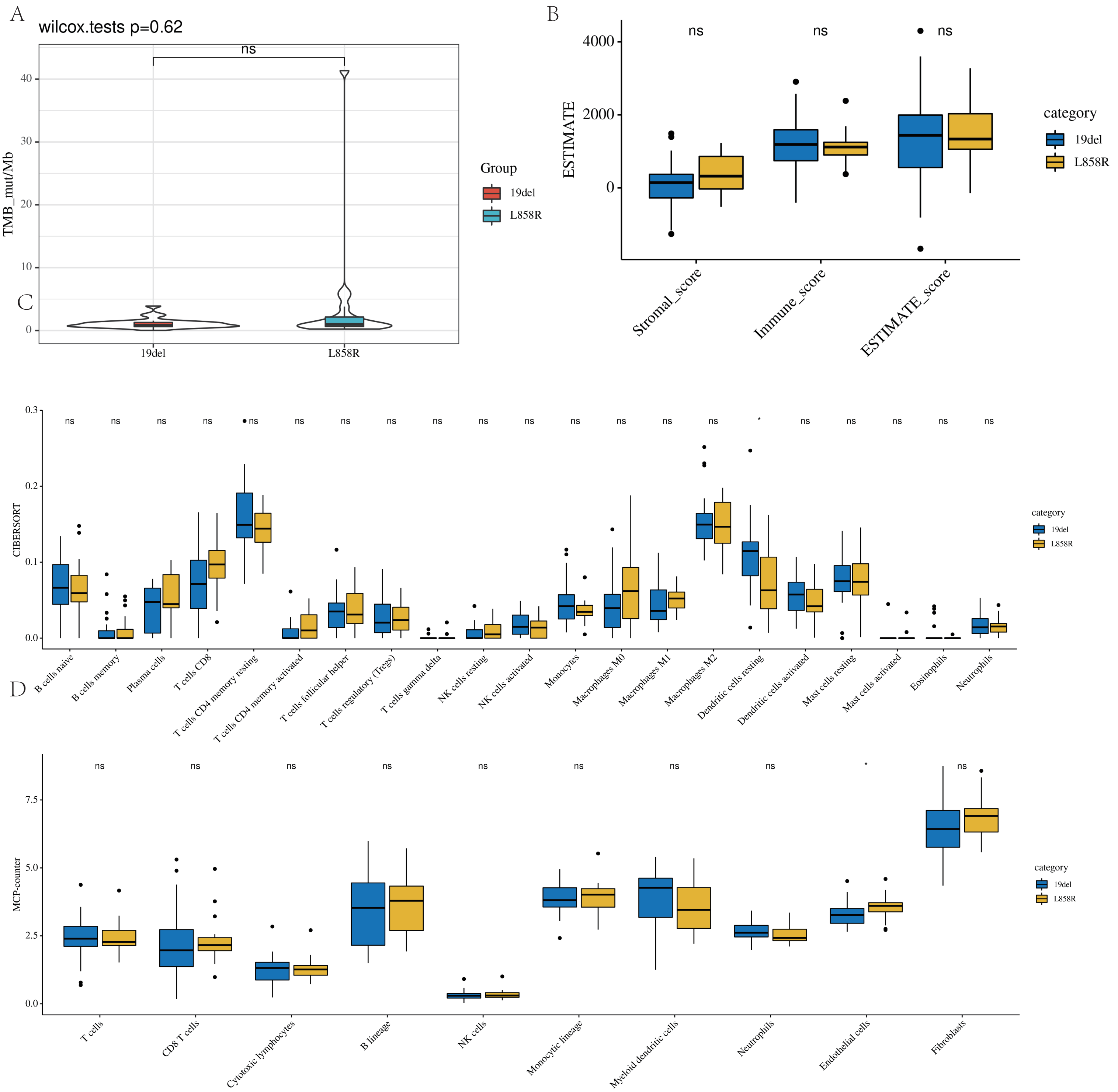

Supplementary Figure 5 Comparison of the tumor-infiltrating lymphocytes between EGFR-mutated group, and KRAS-mutated group. More abundant of CTL and lower of myeloid DC cells were observed in tumor stroma in KRAS mutation versus EGFR mutation patients by MCP-counter (A) as well as the result by CIBERSORT (B). *, **, ***, and **** represent P < 0.05, P < 0.01, P < 0.001 and P < 0.0001, respectively.

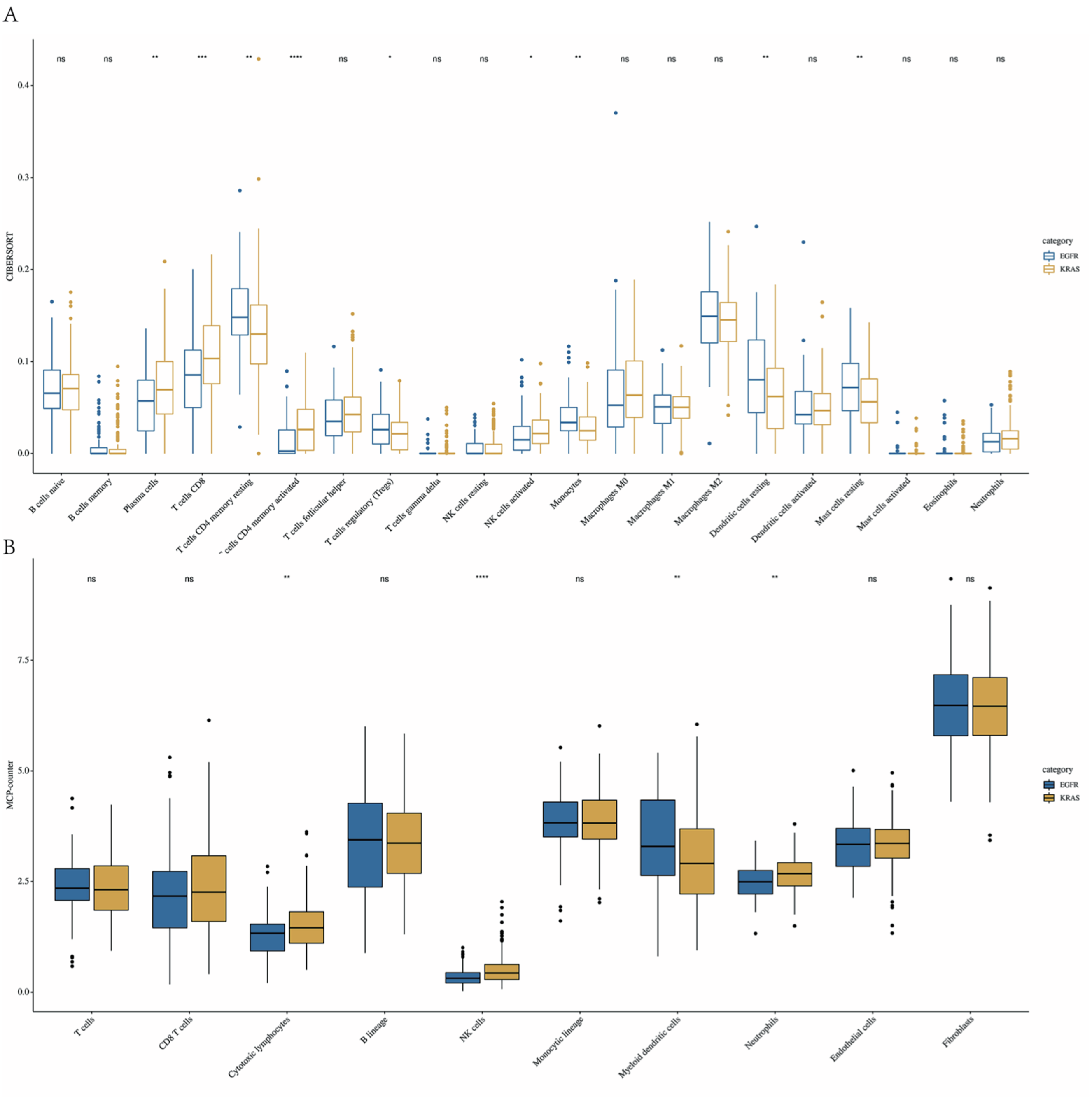

Supplement: Supplementary file 1 [file DataSheet_1.pdf]
