# Supplementary material for: Exploration of the Tumor-Suppressive Immune Microenvironment by Integrated Analysis in EGFR-Mutant Lung Adenocarcinoma
Source: Front Oncol. 2021 May 31;11:591922. doi: 10.3389/fonc.2021.591922 (PMC8200668; doi:10.3389/fonc.2021.591922)
Supplement: Supplementary file 2 [file Table_1.docx]

**Supplementary Table 1 Clinical characteristics of the patients defined by D score**

| Characteristic | Type | Total | High D group | Low D group | Pvalue |
| --- | --- | --- | --- | --- | --- |
| age | <=65 | 234(46.52%) | 117(46.43%) | 117(46.61%) | >0.05 |
|  | >65 | 251(49.9%) | 126(50%) | 125(49.8%) |  |
|  | unknow | 18(3.58%) | 9(3.57%) | 9(3.59%) |  |
| gender | FEMALE | 271(53.88%) | 120(47.62%) | 151(60.16%) | < 0.05 |
|  | MALE | 232(46.12%) | 132(52.38%) | 100(39.84%) |  |
| mutation | EGFR | 76(15.11%) | 19(7.54%) | 57(22.71%) | < 0.05 |
|  | KRAS | 169(33.6%) | 85(33.73%) | 84(33.47%) |  |
|  | others | 258(51.29%) | 148(58.73%) | 110(43.82%) |  |
| stage | Stage I-II | 388(77.14%) | 194(76.98%) | 194(77.29%) | >0.05 |
|  | Stage III-IV | 108(21.47%) | 57(22.62%) | 51(20.32%) |  |
|  | unknow | 7(1.39%) | 1(0.4%) | 6(2.39%) |  |
| T | T1-2 | 438(87.08%) | 217(86.11%) | 221(88.05%) | >0.05 |
|  | T3-4 | 63(12.52%) | 34(13.49%) | 29(11.55%) |  |
|  | unknow | 2(0.4%) | 1(0.4%) | 1(0.4%) |  |
| M | M0 | 339(67.4%) | 165(65.48%) | 174(69.32%) | >0.05 |
|  | M1 | 25(4.97%) | 13(5.16%) | 12(4.78%) |  |
|  | unknow | 139(27.63%) | 74(29.37%) | 65(25.9%) |  |
| N | N0 | 322(64.02%) | 164(65.08%) | 158(62.95%) | >0.05 |
|  | N1-3 | 170(33.8%) | 85(33.73%) | 85(33.86%) |  |
|  | unknow | 11(2.19%) | 3(1.19%) | 8(3.19%) |  |
